# Effect of salinity on growth and biochemical responses of brinjal varieties: implications for salt tolerance and antioxidant mechanisms

**DOI:** 10.1186/s12870-024-04836-9

**Published:** 2024-02-21

**Authors:** Jawaria Jameel, Tauseef Anwar, Saadat Majeed, Huma Qureshi, Ejaz Hussain Siddiqi, Sundas Sana, Wajid Zaman, Hayssam M. Ali

**Affiliations:** 1 Department of Botany, The Islamia University of Bahawalpur (Baghdad-ul-Jadeed Campus), Bahawalpur, 63100 Pakistan; 2 Institute of Chemical Sciences, Bahauddin Zakariya University, Multan, 60800 Pakistan; 3 Department of Botany, University of Chakwal, Chakwal, 48800 Pakistan; 4 Department of Botany, University of Gujrat, Gujrat, 50700 Pakistan; 5 Department of Life Sciences, Yeungnam University, Gyeongsan, 38541 Republic of Korea; 6 Department Department of Botany and Microbiology, College of Science, King Saud University, Riyadh, 11451 Saudi Arabia

**Keywords:** Crop productivity, Salt stress, Oxidative stress, Plant adaptation, Brinjal resilience

## Abstract

Salinity poses significant challenges to agricultural productivity, impacting crops’ growth, morphology and biochemical parameters. A pot experiment of three months was conducted between February to April 2023 in the Department of Botany, The Islamia University of Bahawalpur. Four brinjal (eggplant) varieties: ICS-BR-1351, HBR-313-D, HBR-314-E, and HBR-334-D were selected and assessed for the effects of salinity on various growth and biochemical attributes. The experiment was completely randomized in design with three replicates each. This study revealed that increased salinity significantly reduced the shoot length, root length, and leaf number across all varieties, with maximum adverse effects observed at a 300mM NaCl concentration. Among the tested varieties, ICS-BR-1351 demonstrated superior performance in most growth parameters, suggesting potential salt tolerance. Biochemically, salinity decreased chlorophyll content across all varieties, with the sharpest decline observed at the highest salt concentration. V4 (HBR-334-D) showed a 57% decrease in chlorophyll followed by V3 (HBR-314-E) at 56%, V2 (HBR-313-D) at 54%, and V1 (ICS-BR-1351) at 33% decrease at maximum salt levels as compared to control. Conversely, carotenoid content increased up to -42.11% in V3 followed by V2 at -81.48%, V4 at -94.11%, and − 233% in V1 at 300mM NaCl stress as compared to respective controls. V3 (HBR-314-E) has the maximum value for carotenoids while V1 has the lowest value for carotenoids as compared to the other three brinjal varieties. In addition to pigments, the study indicated a salinity-induced decrease in total proteins and total soluble sugar, whereas total amino acids and flavonoids increased. Total proteins showed a decrease in V2 (49.46%) followed by V3 (36.44%), V4 (53.42%), and V1 (53.79%) at maximum salt concentration as compared to plants treated with tap water only. Whereas, total soluble sugars showed a decrease of 52.07% in V3, 41.53% in V2, 19.49% in V1, and 18.99% in V4 at the highest salt level. While discussing total amino acid, plants showed a -9.64% increase in V1 as compared to V4 (-31.10%), V2 (-36.62%), and V3 (-22.61%) with high salt levels in comparison with controls. Plant flavonoid content increased in V3 (-15.61%), V2 (-19.03%), V4 (-18.27%) and V1 (-27.85%) at 300mM salt concentration. Notably, salinity elevated the content of anthocyanin, lycopene, malondialdehyde (MDA), and hydrogen peroxide (H_2_O_2_) across all varieties. Antioxidant enzymes like peroxidase, catalase, and superoxide dismutase also increased under salt stress, suggesting an adaptive response to combat oxidative damage. However, V3 (HBR-314-E) has shown an increase in anthocyanin at -80.00%, lycopene at -24.81%, MDA at -168.04%, hydrogen peroxide at -24.22%, POD at -10.71%, CAT as-36.63 and SOD as -99.14% at 300mM NaCl stress as compared to control and other varieties. The enhanced accumulation of antioxidants and other protective compounds suggests an adaptive mechanism in brinjal to combat salt-induced oxidative stress. The salt tolerance of different brinjal varieties was assessed by principal component analysis (PCA), and the order of salt tolerance was V1 (ICS-BR-1351) > V4 (HBR-334-D), > V2 (HBR-313-D) > V3 (HBR-314-E). Among the varieties studied, ICS-BR-1351 demonstrated resilience against saline conditions, potentially offering a promising candidate for saline-prone agricultural areas.

## Introduction

Salt stress can become a severe problem for sustainable agriculture practices as it is a huge risk to the agriculture sector in arid and semi-arid regions of the world [1, 2]. Climate change including temperature variations and loss of water is increasing salt levels that affect the growth and productivity of crops [3, 4]. The growth of plants is reduced due to extreme environmental conditions such as water scarcity, high salt levels, heavy metals, and temperature extremes [5, 6]. Environmental stressors hinder plant growth and crop output, which has a significant negative financial impact [7]. It is the need of the hour to get maximum yield from crop plants to feed the increasing population of the world which is expected to be more than 12 billion in 2100 [8, 9]. To meet the growing worldwide need for food, scientists are working to reduce the negative impact of abiotic factors, increase agricultural products, advance sustainability, and ensure food security [10]. On the other hand, the physiological, biochemical, and molecular functions of plants are impaired due to salinity in a way that sustainable agriculture faces serious threats [11]. Salt stress is a complex phenomenon that involves physical, physiological, and ionic imbalances in plants [12]. More than 10% of the total land area is under salt stress making it difficult for crops to grow and survive [13]. Compared to other horticultural crops, vegetable crops are more vulnerable to climate change [14].

Salinity creates adverse conditions for plants osmotic damage, nutritional imbalance, and enzyme action. Salinity-induced oxidative stress affects plant function because nucleic acid and protein are damaged destroying cellular membranes and photosynthetic efficiency resulting in poor growth [15, 16]. The first phase of plant life i.e. seed germination is affected negatively by increasing salt levels around the globe due to higher concentrations of salts in growing media [17]. High level of reactive oxygen species is produced under stress conditions though being stress sensors and signaling molecules, ROS are harmful for plants at all stages of life [18, 19]. Chloroplast is a primary site for the production of ROS as a result carotenoids, chlorophylls, and photosystems fail to perform their functions when the overproduction of ROS occurs [20]. Stress-induced plant water uptake is disturbed and various defense strategies are adapted by the plant to deal with harsh situations [21]. Stress tolerance in plants is mediated by primary and secondary metabolites. The first level of defense is achieved by primary metabolites namely antioxidants. Increased production of antioxidants in stressed plants is essential in scavenging reactive oxygen species that can otherwise affect membrane stability, nucleic acid, and proteins [22, 23]. Vascular plants accumulate proline in response to environmental stress which helps plants grow and stimulates flowering. Identifying and planting salt-tolerant varieties of plants will have a great impact on the economy and food security. The only way to guarantee global food security is to cultivate crops that can withstand salinity [24, 25].

Eggplant is a nutritious vegetable rich in fiber and various minerals, making it a valuable addition to a healthy diet.

It is used as Ayurvedic medicine for the treatment of diabetes, obesity, and hypertension for ages. The nutritional value of brinjal is comparable to tomato plants in terms of minerals and vitamins [26]. Eggplant is a glycophyte that responds to salinity by reducing growth parameters and adjusting physio-biochemical properties, due to this reason it is known as moderately susceptible to salt stress [27]. reported a decline in germination percentage and survival rate of brinjal and tomato plants under salt stress [28]. The purpose of this study is to explore the physiological and biochemical adaptations of four brinjal varieties to combat NaCl stress. The study also revealed the relative salt tolerance response of all four varieties (ICS BR1351, HBR313D, HBR314E and HBR334D) and recommended salt tolerant variety for future cultivation.

## Materials and methods

### Treatment design

To assess the impact of NaCl on four brinjal varieties, a pot experiment was carried out between February-April 2023 at the Islamia University of Bahawalpur, Pakistan. The study area is situated in southern Punjab of Pakistan, where the temperature ranges from 24.5 to 52 °C in summer and in winter between 10.9 and 20.3 °C with semi-arid details. From the University of Agriculture, Faisalabad four varieties of Brinjal (*Solanum melongena* L.) seeds were collected (ICS-BR-1351, HBR-313-D, HBR-314-E and HBR-334-D). The experiment was completely randomized (CRD) in layout with three replicates for each treatment. Seeds were treated with NaOCl for 5 min for surface sterilization. Following that, double distilled water was used to wash the seeds. After that seeds were sown into pots of one-kg size (27.9 × 17.78 cm), filled with 90% loamy soil and 10% compost which were further allowed to germinate under necessary conditions. Soil analyses were also performed before adding soil to the plastic pots (Table 1).

**Table 1:** Soil attributes for brinjal pot experiment

Soil Attributes	Unit	Attribute value	Reference
Soil Depth	cm	0.15	[29]
Texture	-	Loam	
Phosphorus	mg/kg	3.9	[30]
Potassium	mg/kg	126	
Zinc	mg/kg	2.98	[31]
EC	mS/cm	2.3	[32]
pH	-	8.23	[33]

Seedlings were initially cultivated in one-kilogram pots and subsequently transferred to larger five-kilogram pots for continued growth and exposure to salt treatment.

5 kg loamy soil was added to each plastic pot for salt treatment in three replicates and seedlings of 25 days were shifted in them. To prevent abrupt damage to plants, various levels of NaCl were maintained in two consecutive intervals as 0, 50, 100 and 150 mM NaCl to give an amount of 0, 100, 200 and 300mM salt concentration. The first saline treatment was given to brinjal seedlings after 30 days of germination, and the second treatment was given after one weak of the first. With the chosen NaCl levels, irrigation was made using tap water. After one week of both NaCl treatments, brinjal seedlings were harvested for morpho-physiological and biochemical analysis.

### Morphological analysis

After 60 days of sowing, three healthy plants from each pot were selected to analyze the growth and morphology. All brinjal plants of the control group and treatment groups had their shoot length, root length, leaf number, root number, and leaf area measured. The fresh and dry weight of the plant sample was also measured using a digital weighing balance. Dried biomass of the same plant from each sample was also measured after five days of 70 °C oven drying.

### Photosynthetic pigments

Chlorophyll extraction was done with 80% acetone using one-gram leaf sample via homogenization [34]. Acetone was added to make supernatant up to 100 mL. this method was followed with slight changes to measure the photosynthetic attributes of the brinjal plant. Filtered leaf samples were observed at 663, 645 and 480 nm on a Hitachi U-5100 UV-Vis Spectrophotometer (Systronics 128, Japan).

$$Chl\,a \left(\frac{mg}{g}\right)FW=\frac{12.7\times \left({A}_{663}\right)-2.69\times \left({A}_{665}\right)\times V}{1000\times W}$$

$$Chl\,b \left(\frac{mg}{g}\right)FW=\frac{22.9\times \left({A}_{645}\right)-4.68\times \left({A}_{663}\right)\times V}{1000\times W}$$

$$Total\,Chl \left(\frac{mg}{g}\right)FW=\text{C}\text{h}\text{l}\,\text{a}+\text{C}\text{h}\text{l}\,\text{b}$$

$$Carotenoids \left(\frac{\mu g}{mg}\right)FW=\frac{1000\times \left({A}_{470}\right)-1.28 \left(Chl a\right)\times 56.7\left(Chl b\right)}{256\times 0.906}$$

A = Absorbance of chlorophyll extract on specific induced wavelength; V = Final volume of extract in a mixture of 80% acetone; FW = Fresh weight of tissue (mg).

### Total protein estimation

Bradford technique was used to estimate proteins. 0.25 gm of brinjal fresh leaves were mixed in phosphate buffer (pH 7) into 5mL Bradford reagent. The sample was mixed and the absorbance of each sample was measured at 590 nm.

### Total soluble sugar and flavonoid content

The total soluble sugar of the brinjal plant grown under salinity was calculated with slight changes using the anthrone method [34]. 750 µl of anthrone reagent and 50 µl of leaf supernatant were combined, and then the mixture was incubated at 100℃ for 10 min using an Eppendorf thermo mixer (compact 5350). For a short period, reaction tubes were submerged in ice. A blank tube containing 50 µl of 80% ethanol was added to a 750 µl throne reagent and incubated under the same conditions. Upon transferring 150 µl of sample from the test tube to a transparent 96-well microplate, the absorbance of every well was measured at 626 nm. 500 mg of fresh plant leaves were crushed and extract was obtained in ethyl alcohol at 25 °C. Flavonoid content was calculated with the colorimetric method. The calibration curve was created by catechin as standard. At 510 nm spectrophotometer displayed absorbance.

### Total amino acids estimation

150 µl of leaf supernatant was mixed with 75 µl of ninhydrin in a centrifuge tube [35]. Then the mixture was heated at 100 °C for 10 min. 150 µl of 80% ethanol mixed with 75 µl ninhydrin is considered a blank tube. Reaction tubes were kept in ice and 375 µl 95% ethanol was added in every tube. Almost half of the sample was transferred from the assay tube to 96 well microplate and the absorbance of each well was measured at 440, 520, and 570 nm following [36]. Using the calibration curve made with L-proline and L-glycine standard solutions, the free amino acids content was reported as mg of equivalent L- L-proline and L- L-glycine per milliliter.

### Anthocyanin content

Each fully grown brinjal plant’s anthocyanin content was extracted using 1% hydrochloric acid in ethanol at a constant volume. The extract was centrifuged twice and absorbance at 530 nm was measured using a spectrophotometer [37].

### Lycopene estimation

The amount of lycopene was estimated with fewer modifications [38]. The aluminum foil-covered tube was filled with the same volume of hexane and 10 mL leaf extract. The tube spent 15 min being agitated at 180 revolutions per minute in an ice bath. 5 more minutes were spent shaking the materials in tubes containing 3mL of deionized water. The tubes were allowed to settle at room temperature. The Lycopene hexane layer was measured at 503 nm.

$$Lycopene=A\times 31.2/m$$

where A is the absorbance rate and m is the weight of the leaf sample in 10 mL of solvent.

### Determination of H_2_O_2_ content and MDA

Samples weighing (0.5 g) were homogenized in 4 mL of 1% w/v trichloroacetic acid at 4 °C. Homogenates were centrifuged for 20 min at 12,000 rpm. 3.5 mL of 20% TCA containing. 5% TBA was added in 1 mL supernatant and mixed thoroughly. The solution was boiled up to 95 °C for half an hour and quickly submerged in ice. After that optical density of the sample was measured at 532 nm [39].

### Temporal study of antioxidant enzymes

The study investigated the antioxidants enzyme activity of POD, CAT and SOD by implementing respective protocols. A sample treated with distilled water was treated as the control.

One gram of brinjal seedling homogenized in 1 mL of 50 mM (potassium-phosphate) buffer at pH 7. The homogenate was then centrifuged at 12,000 rpm and 4 °C for 15 min.

### Peroxidase assay

Using the Chance and Maehly approach, measuring absorbance at 470 nm every 20 s POD was assessed. Tetra guaiacol was formulated by H_2_O_2_ peroxidation using an electron source and this process was used to test the peroxidase activity. A mixture of 20 mM guaiacol, 40 mM H_2_O_2_ and 50 mM potassium phosphate buffer pH 5 was added to 0.1 mL of sample enzyme extract [40].

### Catalase assay

The reaction mixture (3 mL) comprising 1mL enzyme extract, 5.9 mM H_2_O_2_ and 50 mM phosphate buffer were evaluated. The change in absorbance at 240 nm due to H_2_O_2_ intake was measured to determine CAT activity [40].

### Superoxide dismutase assay

Using the ability of SOD to stop the photochemical reduction of nitro blue tetrazolium NBT was determined. The optical density was measured at 560 nm. The reaction mixture has 100 mM of EDTA, 13 mM of methionine, 75 mM of NBT, 2 mM of riboflavin, 50 mM of sodium phosphate buffer (pH 7.8), and 100 mL of enzyme extract [41].

### Statistical analysis

All data of the experiments was represented as the mean value of three replicates. Graphs were made by Origin 2021 Pro software. A two-way factorial analysis was executed via paired comparisons. Fisher’s LSD test was used to compare the treatment means, with a significance level of *p* < 0.05. Principle component analysis was also made for exploratory data description.

## Results

### Effect of salt stress on the growth of plant

Salt treatment affected the morphological attributes of plants negatively as shoot length, root length, number of leaves and number of roots, plant fresh weight, plant dry weight, and leaf area showed significant reduction at all salt levels in comparison with control plants. Data representing the shoot length of brinjal plants revealed that shoot length decreased considerably with growing levels of salt stress. V-1 (ICS-BR-1351) showed maximum value for SL, RL, PFW, PDW, LA and number of leaves, at maximum (300mM NaCl) treatment followed by V-4 (HBR-334-D). Better growth performance indicated salt tolerance in V-1 and V-4, however, V-2 and V-3 have the lowest values of root length, shoot length, number of leaves, and plant fresh and dry weight at salt treatment.

**Fig. 1:**
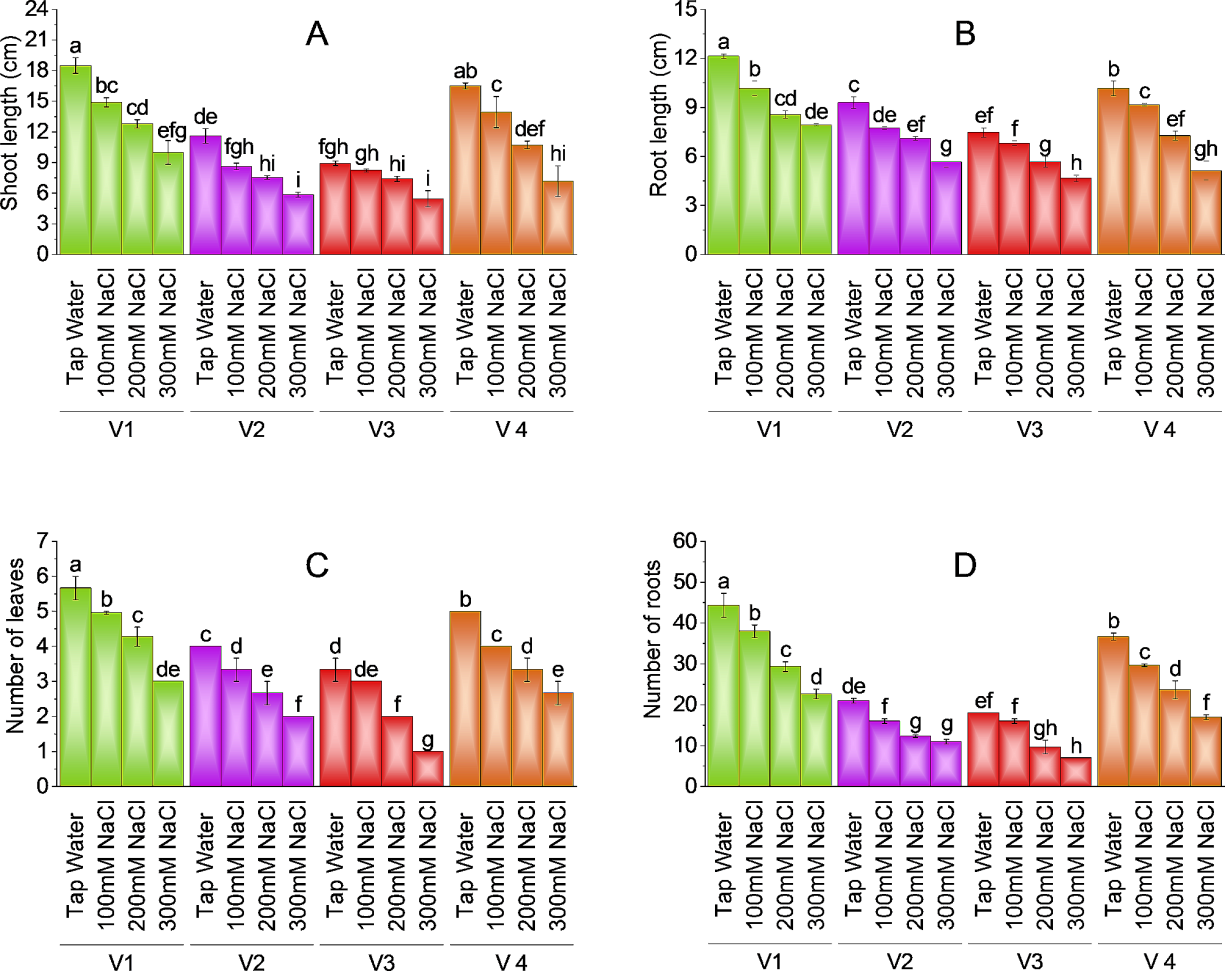
Effect of different levels of salinity on shoot length (**A**), root length (**B**), number of leaves (**C**), and number of roots (**D**) of brinjal. Bars are means of three replicates ± SE. Different letters on bars showed significant changes at *p* ≤ 0.05 compared using Fisher’s LSD

In terms of salt damage, the greatest reduction of shoot length was observed at 300mM NaCl treatment in all the varieties. V-3 showed (38.81%), V-1 (45%), V-2 (49.43%) and V-4 (56.42%) reduction in shoot length at a maximum salt concentration as compared to control plants. At 100mM and 200mM salt treatment maximum decrease in shoot length was observed in V-2 (25.57%) and (35.06%) followed by V-1 (19.46%) and (30.81) respectively. The maximum value of shoot length is reported in V-1 control plants as (18.50 cm). V-3 (HBR-314-E) had the lowest value of shoot length (5.47 cm) at maximum salt concentration showing susceptibility to salt stress, followed by V-2 (HBR-313-D) with (5.87 cm) at the same concentration (Fig. 1A).

A significant change in root length was observed in different varieties of brinjal plants grown in salt stress. Brinjal V-3 (HBR-314-E) has the lowest value of root length (4.67 cm) in comparison to other varieties. V1 (ICS-BR-1351) showed the highest value of root length (12.13 cm) followed by V-4 (HBR-334-D) at 10.17 cm and V-2 (HBR-313-D) at 9.30 cm respectively. While considering salt toxicity all varieties showed a decrease in root length. The maximum decline was reported at the highest salt concentration of 300mM. Plant V-1 (ICS-BR-1351) has a 34% decrease in root length followed by V-3 (HBR-314-E) at 37.50%, V-2 (HBR-313-D) at 39.07%, and V-4 (HBR-334-D) has 49.50% decline in root length at 300mM salt concentration (Fig. 1B). Maximum reduction in root length at 100mM and 200mM salt treatment was observed as 16.21% and 29.40% in V-1, 16.85% and 23.66% in V-2, 8.93% and 24.11% in V-3 and 9.84% and 28.52% in V-4 respectively.

The number of leaves in brinjal plants showed variability in a way that a maximum number of leaves were observed in V-1 (ICS-BR-1351) at 5.67 on average followed by V-4 (HBR-334-D) and V-2 (HBR-313-D). V-3 (HBR-314-E) has the lowest number of leaves (1 leaf per plant) as compared to all varieties. Maximum decline in leaf number is reported at the highest salt concentration. V-3 (HBR-314-E) has a 70% decline in leaf number followed by V-2 (HBR-313-D) with a 50% decrease and V-1 (ICS-BR-1351) with 47.06%, V-4 (HBR-334-D) as 46.66% decrease as compared to control (Fig. 1C).

Results showing the number of roots per plant revealed that V-3 (HBR-314-E) has the lowest number of roots (7 per plant) in comparison to all other varieties. V-1 (ICS-BR-1351) has the highest value for the number of roots (44.33) on average followed by V-4 (HBR-334-D) at (36.67). Salt toxicity decreased the number of roots significantly. Plants had a slight decrease in the number of roots at 100mM salt concentration however more decrease is observed at 200-300mM NaCl treatment. V-3 (HBR-314-E) has 61.11%, V4 (HBR-334-D) has 53.63%, V-1 (ICS-BR-1351) has 48.87%, and V-2 (HBR-313-D) has 47.62% decline in number of roots at 300mM salt treatment as compared to control (Fig. 1D).

**Fig. 2:**
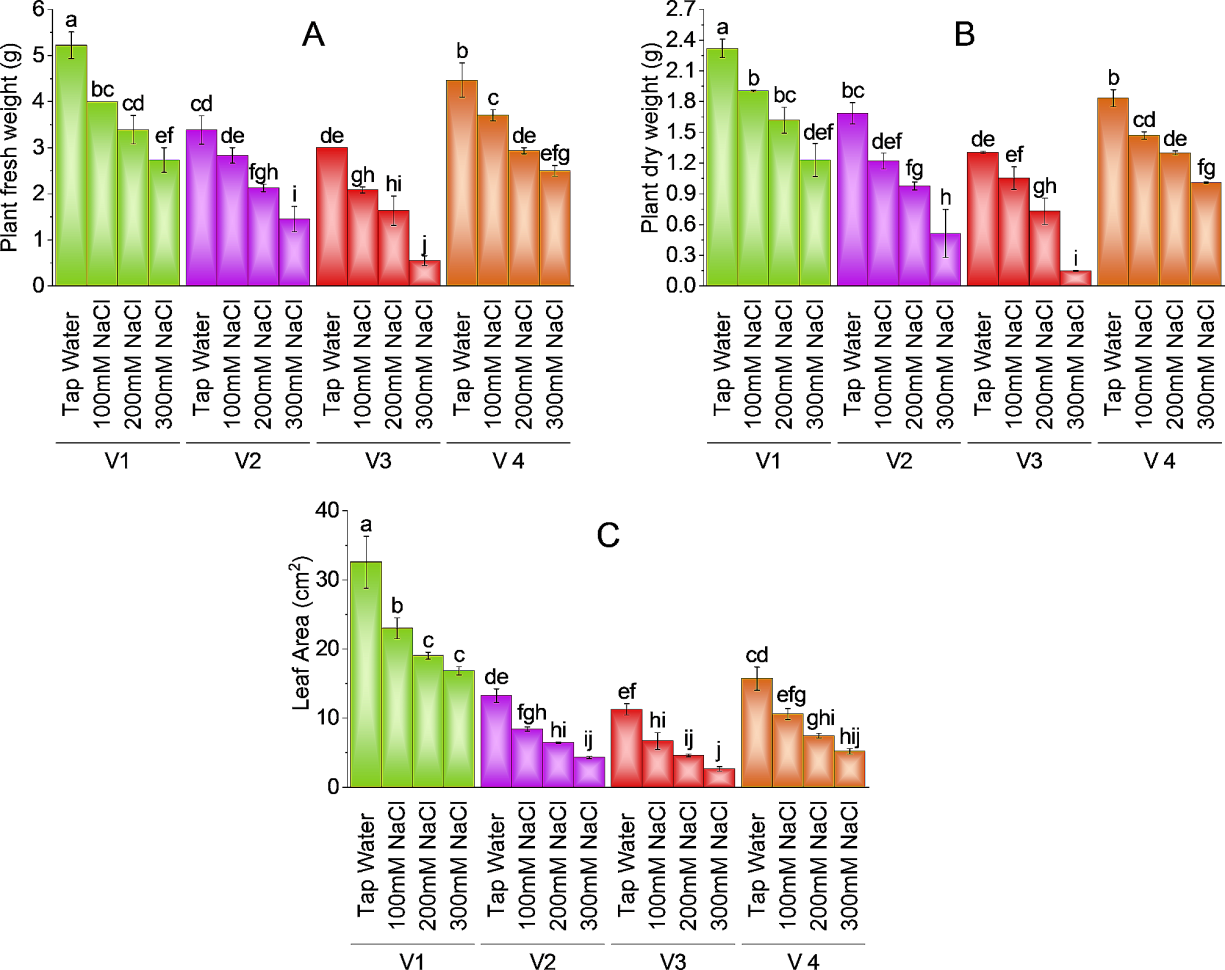
Effect of different levels of salinity on plant fresh weight (**A**), plant dry weight (**B**), and leaf area (**C**) of brinjal. Bars are means of three replicates ± SE. Different letters on bars showed significant changes at *p* ≤ 0.05 compared using Fisher’s LSD

While considering plant fresh and dry weight study revealed that all brinjal varieties showed a reduction in fresh and dry matter at all levels (100mM, 200mM, and 300mM) of salt. Plant V-3 (HBR-314-E) has the lowest value of biomass (0.55 g and 0.15 g) as compared to other varieties. V-1 (ICS-BR-1351) has a maximum value of fresh and dry weight (5.22 g and 2.32 g) followed by V-4 (HBR-334-D) and V-2 (HBR-313-D) respectively. In terms of salt stress brinjal V-1 (ICS-BR-1351) showed 47.67% and 46.98% decline in fresh and dry weight at 300mM salt concentration as compared to control plants. V-2 (HBR-313-D) has a 57.09% reduction in fresh weight and 69.57% decrease in dry weight at maximum salt concentration as compared to control while V-3 (HBR-314-E) has an 81.67% decrease in fresh and 88.75% decrease in dry weight at 300mM NaCl treatment as compared to control (Fig. 2A & B).

The leaf area of the plant is also negatively affected by salt stress in a way that significant reduction is observed in all varieties at various levels of salt stress. The maximum value of leaf area (32.57cm^2^) is observed in V-1 (ICS-BR-1351) followed by V-4 (HBR-334-D) as (15.72cm^2^), the minimum value of leaf area (23.67cm^2^) is observed in V-3 at the highest salt level. V-1 (ICS-BR-1351) showed a 48.31% decline in leaf area at 300mM salt concentration as compared to control plants. V-4 (HBR-334-D) had 66.91%, V-2 (HBR-313-D) 67.51% reduction and V-3 (HBR-314-E) had a 76.33% decline in leaf area at maximum salt concentration as compared to control (Fig. 2C). V-1 showed a 29.38% decline at 100mM and 41.56% at 200mM salt stress while V-3 showed a 40.53% decline in leaf area at100mM, and 59.17% at 200mM salt treatment as compared to the control.

### Photosynthetic and accessory pigments

**Fig. 3:**
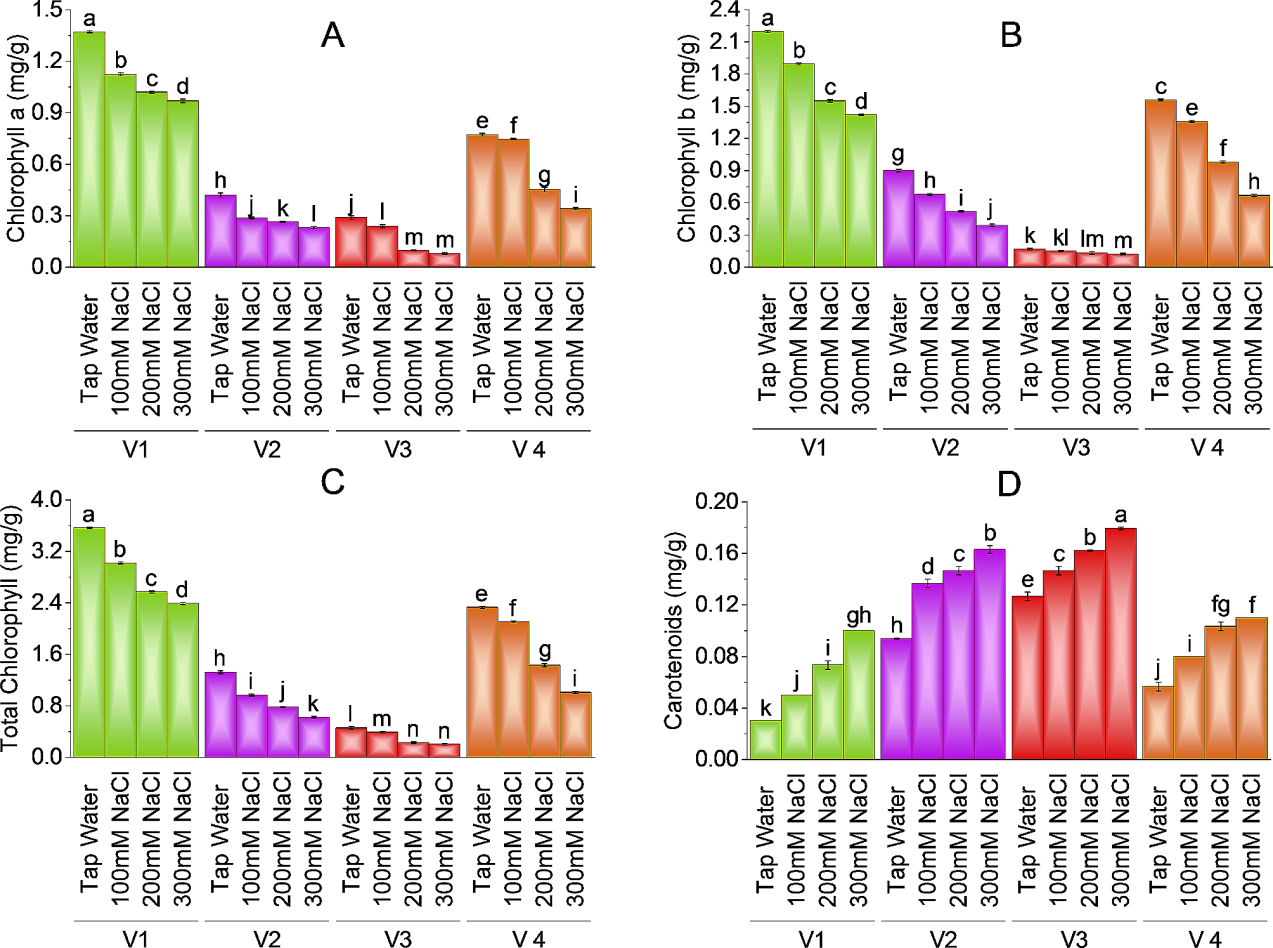
Effect of different levels of salinity on chlorophyll a (**A**), chlorophyll b (**B**), total chlorophyll (**C**), and carotenoids (**D**) of brinjal. Bars are means of three replicates ± SE. Different letters on bars showed significant changes at *p* ≤ 0.05 compared using Fisher’s LSD

The response of plants to salt stress at the photosynthetic level is crucial to estimating the mechanism of salt-sensitive and salt-resistant varieties. V-1 (ICS-BR-1351) has a maximum value of chlorophyll (3.57 mg/g) followed by V4 (HBR-334-D and V2 (HBR-313-D). Brinjal variety V-3 (HBR-314-E) has the lowest value (0.20 mg/g) of chlorophyll in comparison with the other three varieties. All brinjal varieties showed significant reduction in chlorophyll a, b, and total chlorophyll at different levels of salt stress. V-1 showed 18% and 25.5% decrease in chlorophyll an at 100mM and 200mM NaCl treatment respectively as compared to control, whereas chlorophyll b showed 13.81% and 29.44% decline as compared to control on the same salt level in V1. As NaCl concentration is increased chlorophyll level decreases and maximum reduction is observed at the highest salt concentration as compared to control. V-1 (ICS-BR-1351) showed a 29.20% reduction in chlorophyll a, 35.36% decrease in chlorophyll b and 32.99% decline in total chlorophyll content at the highest salt treatment as compared to control. V2 (HBR-313-D) has a 45.24% decrease in chlorophyll a, 56.67 loss in chlorophyll b and 53.03% decrease in total chlorophyll, V3 (HBR-314-E) has 72.41% decline in chlorophyll a 26% loss in chlorophyll b pigment and 55.47% in total chlorophyll at maximum salt stress as compared to control. Plant V4 (HBR-334-D) showed 56.03% loss of chlorophyll a pigment, 57.17% decrease in chlorophyll b and 56.65% decline in total chlorophyll at 300mM salt concentration as compared to control (Fig. 3A, B & C). In the case of carotenoids, plants showed an increase with increasing levels of salt stress. Plant V3 (HBR-314-E) has a maximum value (0.18 mg/g) of carotenoids followed by V2 (HBR-313-D) as (0.16 mg/g) and V4 (HBR-334-D) as (0.11 mg/g) (Fig. 3, D). Carotenoid content showed a -42.11% increase in V3 followed by a -81.48% increase in V2, -94.11% in V4, and − 233.33% in V1 at maximum salt level as compared to control. V-3 showed − 15.79% and − 26.32% increase in carotenoid content at 100mM and 200mM as compared to control plants.

### Total proteins, total soluble sugar, flavonoids, and total amino acids

**Fig. 4:**
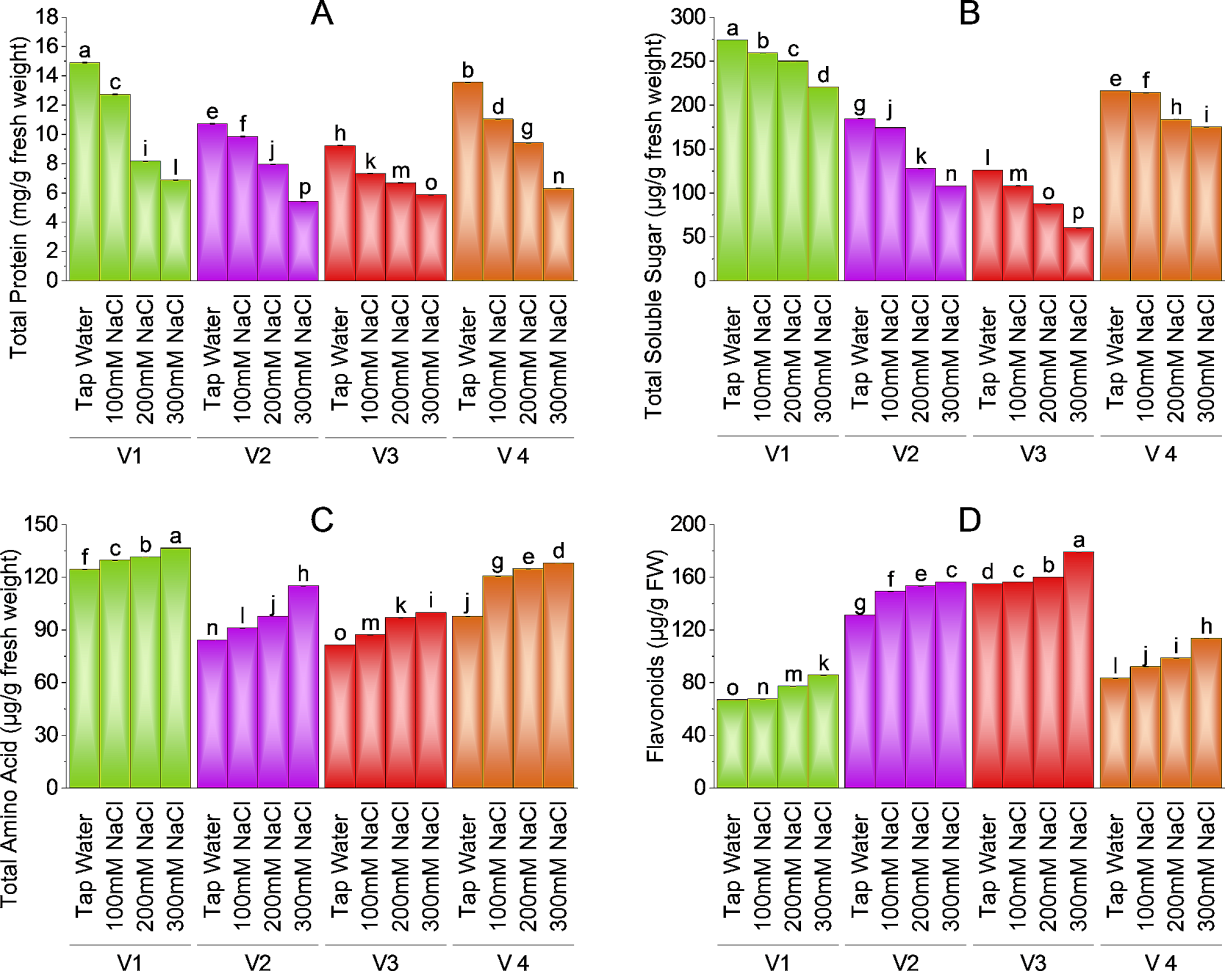
Effect of different levels of salinity on total protein (**A**), total soluble sugar (**B**), total amino acid (**C**), and flavonoids (**D**) of brinjal. Bars are means of three replicates ± SE. Different letters on bars showed significant changes at *p* ≤ 0.05 compared using Fisher’s LSD

Data regarding total protein showed a significant decrease with various salinity levels in all brinjal varieties. V-4 (HBR-334-D) and V1 (ICS-BR-1351) both faced a 54% reduction in protein content as compared to control plants. V-2 has a 50% reduction in protein concentration and V-3 has a 36% decline in protein value as compared to control plants. V-1 (ICS-BR-1351) has the highest value (14.90 mg/g) of total protein in the case of control plants followed by V-4 (HBR-334-D) at 13.55 mg/g, V-2 (HBR-313-D) as 10.72 mg/g and V-3 (HBR-314-E) as 9.24 mg/g compared to salt-treated plants. The minimum value of total proteins (5.87 mg/g) is noted in V-3 at the highest salt level. V-3 showed 20.71% and 27.60% reduction in total proteins at 100 and 200mM salt stress respectively as compared to control plants (Fig. 4A).

### Anthocyanin, Lycopene, MDA and H_2_O_2_ content

Total soluble sugar (TSS) of brinjal varieties is also affected in a similar way as total protein at various levels of salt stress. V-1 (ICS-BR-1351) has the highest value (273 µg/g) of TSS in the case of control plants followed by V-4 (HBR-334-D), V2 (HBR-313-D) and V3 (HBR-314-E) as compared to salt treated plants. A minimum value (60.32 µg/g) of TSS is observed in V-3 at 300mM salt treatment. V-1 has a slight reduction (8.69%) in TSS at 100 mM NaCl treatment as compared to control and a 19.49% decline in TSS at 300mM salt stress as compared to control. V-2 showed a 5.61% decline at 100mM and a 41.53% decrease at 300mM NaCl treatment as compared to the control. V-3 (HBR-314-E) showed a 14%, 30.60%, and 52.07% decline in TSS at 100, 200, and 300mM salt stress as compared to control plants. V-4 (HBR-334-D) had 14.98% and 18.99% reduction at 200 and 300mM salt concentrations respectively (Fig. 4B).

While considering the total amino acids content in the brinjal plant all varieties showed an increase as the salt concentration exceeded. V-1 (ICS-BR-1351) has a maximum value (136.50 µg/g) of amino acid content followed by V-4 (HBR-334-D) as 128.14 µg/g, V-2 (HBR-313-D) as 115 µg/g and V-3 (HBR-314-E) as 81.45 µg/g. V-2 (HBR-313-D) has − 36.62% increase in amino acid, followed by V4 (HBR-334-D) as -31.10%, V-1 (ICS-BR-1351) has − 9.64% increase in amino acid at maximum salt concentration, V3 showed − 22.61% more amino acid at 300mM salt stress as compared to control (Fig. 4C). V-3 has a -6.93% and − 19.05% increase in amino acid content at 100 and 200mM salt treatment as compared to control plants.

The flavonoids content of the plant showed variable values as the maximum content was shown by V-3 (HBR-314-E) at the highest salt level 179.22 µg/g followed by V-2 (HBR-313-D) as 156.29 µg/g and V-4 (HBR-334-D) as 113.66 µg/g and V-1 (ICS-BR-1351) as 85.54 µg/g. V-3 has the maximum value of flavonoids at the highest salt concentration. V-1 (ICS-BR-1351) showed the lowest value for flavonoid content in comparison to other varieties. However, all varieties showed a significant increase in flavonoids as salt levels increased. V-4 showed a -36.39% increase, followed by V-1 (-27.85%), V-2 (-19.03%), and V-3 (-15.61%) increase in flavonoids at maximum salt concentration as compared to control (Fig. 4D).

Brinjal varieties showed an increase in anthocyanin concentration with increasing levels of salt. V-3 (HBR-314-E) had a maximum value (0.03µmol/mL) of anthocyanin followed by V-2 (HBR-313-D) and V-4 (HBR-334-D as 0.02 µmol/mL and V1 (ICS-BR-1351) as 0.01µmol/mL at 300mM salt level. V-2 has a -20% increase V3 has − 80.00% and V-4 and V-1 have − 100% increase in anthocyanin at 300mM NaCl treatment as compared to control plants.

**Fig. 5:**
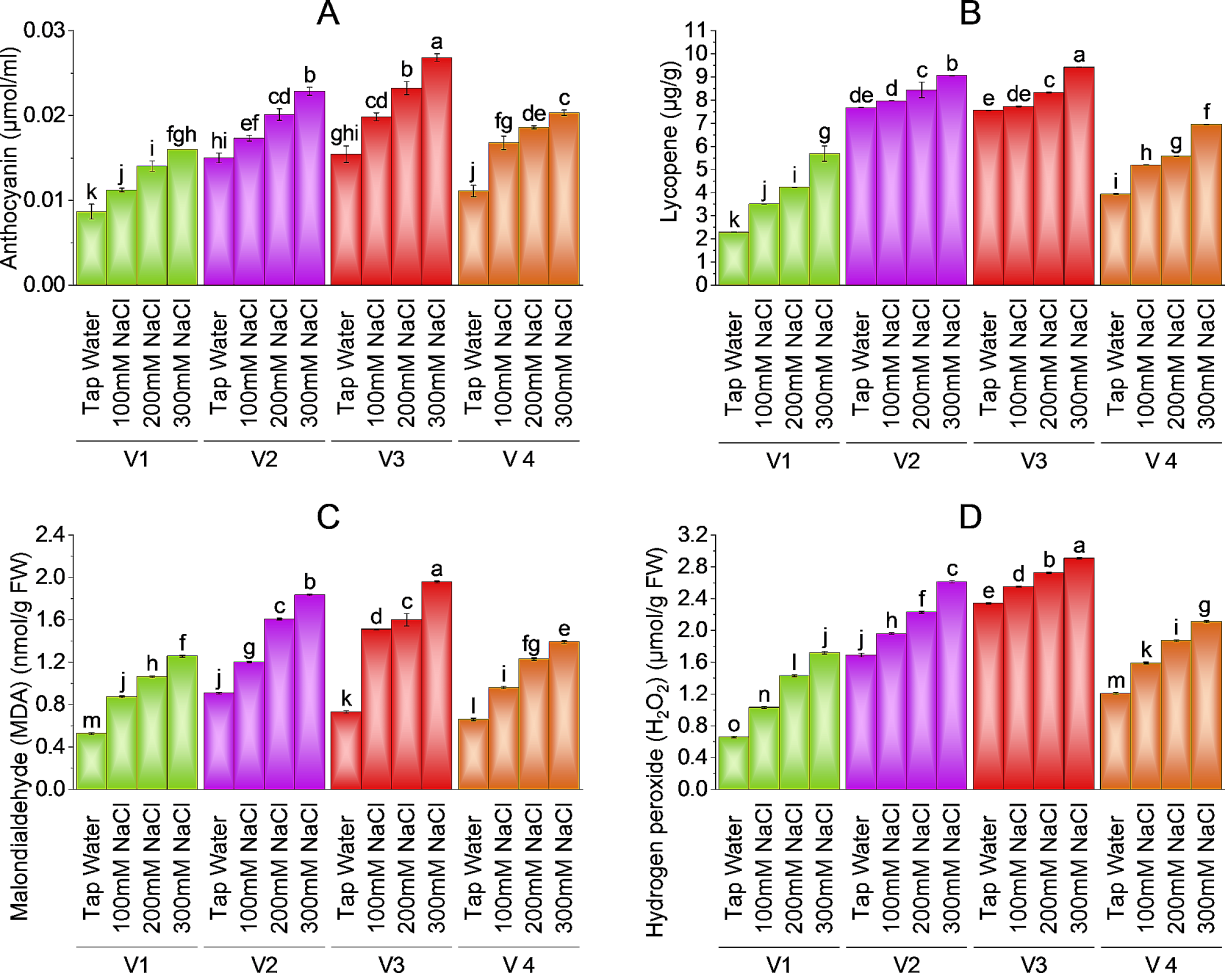
Effect of different levels of salinity on anthocyanin (**A**), lycopene (**B**), MDA (**C**), and H_2_O_2_ (**D**) of brinjal. Bars are means of three replicates ± SE. Different letters on bars showed significant changes at *p* ≤ 0.05 compared using Fisher’s LSD

The Lycopene content of brinjal varieties also showed a significant increase with increasing levels of salt. V-3 (HBR-314-E) and V-2 (HBR-313-D) have a high value of lycopene namely 9.42 µg/g and 9.06 µg/g as compared to the other two varieties. V-3 has − 2.38% and 10.33% increase in lycopene at 100 and 200mM salt treatment as compared to control plants. V-1 (ICS-BR-1351) has the lowest value of lycopene in control plants which increased with increasing levels of salt. V-3 has − 24.81%, V-2 has − 17.98%, and V-4 has − 76.39% increase in lycopene as compared to control. V-1 increased the amount by more than doubled − 148% as compared to control plants (Fig. 5A, B, C & D).

Malondialdehyde and Hydrogen peroxide showed similar values in all brinjal varieties. All varieties showed an increase in MDA and H_2_O_2_ with increasing levels of salinity. V-3 (HBR-314-E) has maximum values (1.96nmol/g) and (2.91µmol/g) for MDA and H_2_O_2_ respectively at maximum salt levels as compared to control plants. Control plants of V-1 (ICS-BR-1351) had less value MDA (0.53nmol/g) and H_2_O_2_ (0.66µmol/g). V-1 showed − 138% and − 160.61% increases V-2 showed − 102.21% and − 54.64%, V-3 showed − 168% and − 24.22%, and V-4 showed − 110% and − 74.89% increases in MDA and H_2_O_2_ at highest salt stress as compared to control plants. V-1 has − 65.82% and − 101.90% increase at 100 and 200mM salt treatments while V-3 has − 106.85% and 119.18% increase in MDA on the same salt treatment as compared to control plants.

### Antioxidants

**Fig. 6:**
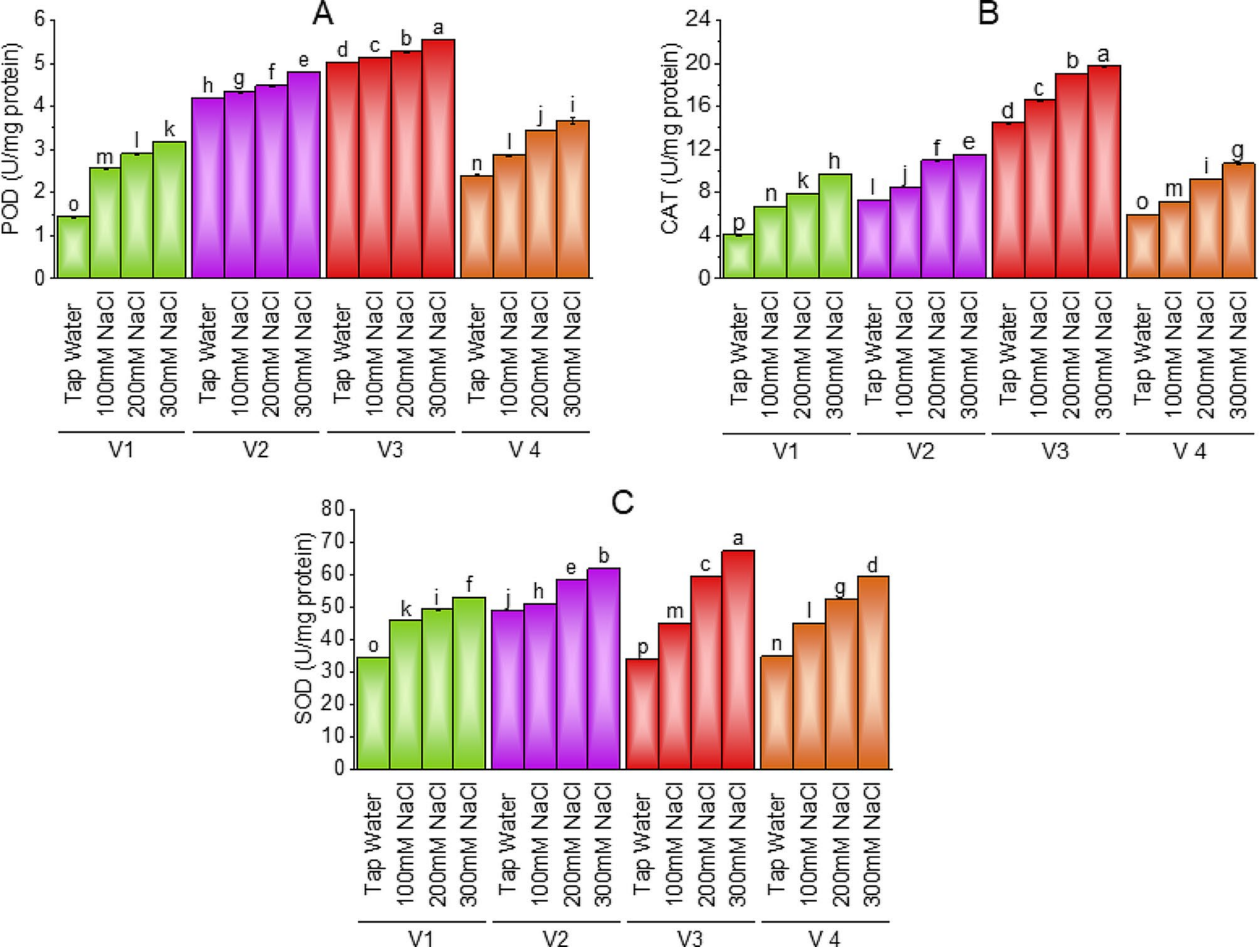
Effect of different levels of salinity on POD (**A**), CAT (**B**) and SOD (**C**) of brinjal. Bars are means of three replicates ± SE. Different letters on bars showed significant changes at *p* ≤ 0.05 compared using Fisher’s LSD

All salt-treated plant varieties had greater value for antioxidants (peroxidase, catalase, and superoxide dismutase) as compared to control plants. The value of POD, CAT, and SOD showed maximum value in the case of V-3 (HBR-314-E) as 5.55U/mg, 19.71U/mg 67.36 U/mg, of POD, CAT, and SOD respectively followed by V-2 (HBR-313-D) and V-4 (HBR-334-D). V-1 (ICS-BR-1351) showed minimum value as compared to other varieties yet at the highest salt levels plants have a maximum increase in antioxidants as compared to plants treated with tap water. Brinjal V-1 (ICS-BR-1351) showed − 121%, V-2 (HBR-313-D) -14.41%, V-3 (HBR-314-E) -10.71% and V-4 (HBR-334-D) showed − 53.41% increase in peroxidase at a maximum salt concentration as compared to control plants. The case of the catalase plant showed a 41.90% increase in V-1, -58.93% in V-2, -36.63% in V-3, and − 80.53% increase in V-4 at 300mM salt stress as compared to control. Superoxide dismutase also had an increase in a similar way that V-1 showed − 54%0.61%, V-2 showed − 26.19%, V-3 showed 99.14% and V-4 showed a -70.33% increase as compared to control plants (Fig. 6A, B, C & D). V-3 showed − 32.33% and − 76.11% increase in SOD, -2.20% and − 5.06% increase in the case of POD, and − 14.44 and − 31.73% increase in CAT at 100 and 200mM NaCl stress as compared to control.

### Cluster analysis

**Fig. 7:**
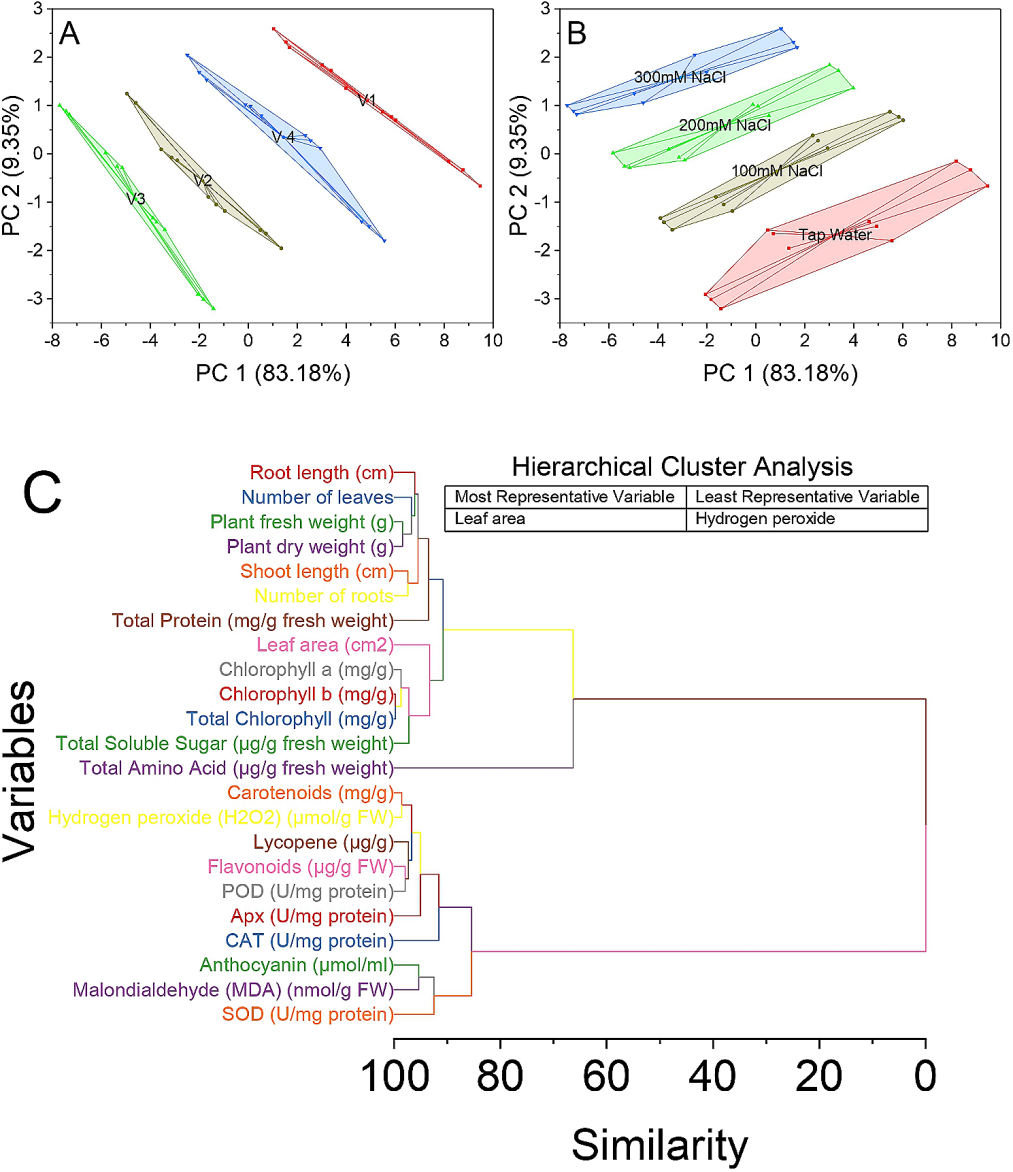
Cluster plot convex hull for varieties (**A**), salinity levels (**B**), and hierarchical cluster analysis for studied attributes (**C**)

The cluster plot convex hull analysis yielded meaningful results, providing insights into the clustering pattern of the varieties in a two-dimensional space. The first principal component (PC 1) explained 83.18% of the total variance, while the second principal component (PC 2) accounted for 9.35% of the total variance. The varieties were labeled as V1, V2, V3, and V4. Examining the scores assigned to each variety in PC 1 and PC 2 revealed their positions within the two-dimensional plot. V-1 displayed scores of 9.47431 in PC 1 and − 0.66165 in PC 2. Similarly, V1 exhibited scores of 8.76702 in PC 1 and − 0.33451 in PC 2, and 8.18836 in PC 1, and − 0.15229 in PC 2. The remaining scores for V1 in PC 1 and PC 2 followed a similar pattern. Additionally, V-2 demonstrated scores of 1.34915 in PC 1 and − 1.95568 in PC 2, 0.72037 in PC 1 and − 1.65042 in PC 2, and 0.49082 in PC 1 and − 1.57742 in PC 2. The scores for V2 in PC 1 and PC 2 continued similarly. Similarly, V-3 displayed scores of -1.42462 in PC 1 and − 3.20469 in PC 2, -1.82956 in PC 1 and − 3.01223 in PC 2, and − 2.05303 in PC 1 and − 2.90919 in PC 2. The scores for V-3 in PC 1 and PC 2 followed a similar pattern. Furthermore, V-4 exhibited scores of 5.56143 in PC 1 and − 1.79578 in PC 2, 4.93852 in PC 1 and − 1.50127 in PC 2, and 4.63402 in PC 1 and − 1.40314 in PC 2. The scores for V-4 in PC 1 and PC 2 continued in a similar fashion (Fig. 7A).

The tap water treatment exhibited scores of 9.47431 in PC 1 and − 0.66165 in PC 2, while the second instance of tap water had scores of 8.76702 in PC 1 and − 0.33451 in PC 2. The third Tap Water treatment displayed scores of 8.18836 in PC 1 and − 0.15229 in PC 2. The scores for Tap Water in PC 1 and PC 2 followed a similar pattern. Similarly, the 100mM NaCl treatment demonstrated scores of 6.02135 in PC 1 and 0.69632 in PC 2, 5.84249 in PC 1 and 0.76579 in PC 2, and 5.47651 in PC 1 and 0.87129 in PC 2. The scores for 100mM NaCl in PC 1 and PC 2 continued similarly. The 200mM NaCl treatment exhibited scores of 3.98263 in PC 1 and 1.36242 in PC 2, 3.37802 in PC 1 and 1.72399 in PC 2, and 3.01913 in PC 1 and 1.84025 in PC 2. The scores for 200mM NaCl in PC 1 and PC 2 followed a similar pattern. Furthermore, the 300mM NaCl treatment displayed scores of 1.69011 in PC 1 and 2.2023 in PC 2, 1.53494 in PC 1 and 2.31498 in PC 2, and 1.03176 in PC 1 and 2.59303 in PC 2. The scores for 300mM NaCl in PC 1 and PC 2 continued in a similar way (Fig. 7B).

For instance, variables 10 and 11, representing chlorophyll b and total chlorophyll content, respectively, exhibited a similarity value of 0.26271, indicating a high level of similarity. Similarly, variables 9 and 26, representing chlorophyll a and an unspecified variable, displayed a similarity value of 1.05668. Furthermore, variables 14 and 15, representing carotenoids and hydrogen peroxide (H_2_O_2_) levels, demonstrated a similarity value of 1.45271, suggesting a strong relationship between these two variables. Variables 3 and 4, indicating plant fresh weight and plant dry weight, respectively, exhibited a similarity value of 1.62565, indicating their close association. Additionally, variables 17 and 18, representing flavonoids and POD (peroxidase) activity, respectively, showed a similarity value of 2.0912, suggesting a potential relationship between these two variables. Variables 5 and 6, indicating shoot length and the number of roots, respectively, exhibited a similarity value of 2.61226, indicating their close association. Moreover, variables 16 and 29, representing lycopene and an unspecified variable, displayed a similarity value of 0.6053, indicating a moderate level of similarity. Variables 12 and 35, indicating total soluble sugar and an unspecified variable, exhibited a similarity value of 2.76946, suggesting a relationship between these variables (Fig. 7C).

## Discussion

### Growth and morphology

Soil salinity inhibits crop growth and it is alarming for food security as crop plants show a negative impact when grown under stressed conditions [41]. In our study, the brinjal crop demonstrated how stress varies depending on salt concentration and variety. V-1 (ICS-BR-1351) showed the highest growth rate followed by V-4 (HBR-334-D) and V-2 (HBR-313-D). V-3 (HBR-314-E) has the lowest value for all morphological parameters in comparison to other varieties. Based on root and shoot growth V-1 appeared salt tolerant as compared to other varieties. Salt-stressed plants exhibit stunted growth and less development at the seedling stage indicating survival problems [42]. As the growth status of a plant is reflected in terms of morphology our study revealed the impact of salt stress that caused a reduction in the growth parameters of plants in terms of root and shoot length, leaf area, leaf number, and fresh and dry weight of all varieties. Earlier stages of plant life are more prone to salt stress. Cell division and cell elongation are affected as a result root length and shoot length tend to decrease as compared to control plants. All varieties showed reduction in growth parameters with salt treatment and maximum reduction is observed at 200 to 300mM NaCl treatment. Similar results are reported in rice plants where variety showed a reduction in root and shoot parameters when grown under salt stress [43]. A significant decrease in germination and survival of brinjal is reported due to the toxic effects of Na^+^ and Cl^−^ that also limit plant height and carbon assimilation [44]. Similar results have been reported by [45] in maize under salt stress where the crop showed a decrease in root and shoot growth and a decrease in several leaves also. Ion toxicity, osmotic imbalance, stunted plant growth, and deficiency in nutrient absorption occur in wheat grown in salt stress [46] It was also concluded that salt injury in plants is obvious in terms of decline in shoot growth, reduced leaf area, and decreased root zone [47]. A decrease in shoot branches, plant fresh and dry weight, and relative water content is reported in kiwi fruit under the influence of salt stress [48]. The deleterious impact of sodium and chloride ions is observed in brinjal grown under a soil-less system as well where a fewer number of leaves, yellowing of leaves due to salt, and decreased shoot growth are reported. Salinity affects plant growth, stem reduction, and low photosynthesis due to reduced leaf area in tomato and cucumber plants [49].

### Photosynthetic and accessory pigments

The response of plants to salt stress at the photosynthetic level is crucial. Pigment content is an estimation of salt sensitivity and salt resistance mechanism of plant varieties. Plant variety 1 (ICS-BR-1351) has a maximum value of chlorophyll followed by V4 (HBR-334-D and V2 (HBR-313-D). Brinjal variety 3 (HBR-314-E) has the lowest value of chlorophyll in comparison with the other three varieties. Reduction in chlorophyll content due to pigment disturbance occurs under the influence of salt stress in maize plants [50]. In our findings, all brinjal varieties showed a significant reduction in chlorophyll a, b, and total chlorophyll at different levels of salt stress. When NaCl concentration is increased chlorophyll level decreases and maximum reduction is observed at the highest salt concentration as compared to control. When there is a loss of chlorophyll synthesis in plants photosynthetic activity of light and dark reactions is also decreased and thus salt disrupts carbon fixation and product synthesis too [51]. As salt causes reduced leaf area photosynthetic pigments are also affected because fewer leaves have more chlorophyll but a minute contribution in photosynthesis [52]. In the case of carotenoids, plants showed an increase with increasing levels of salt stress. Plant V3 (HBR-314-E) has the maximum value of carotenoids followed by V2 (HBR-313-D) and V4 (HBR-334-D). the carotenoids increase salt stress because they have a role in scavenging reactive oxygen species and also protect chloroplast membrane via the production of heat [53].

### Total proteins, total soluble sugar, flavonoids, and total amino acids

Data regarding total protein showed a significant decrease with various salinity levels in all brinjal varieties. Plant variety 1 (ICS-BR-1351) has the highest value of total protein in the case of control plants followed by V4 (HBR-334-D), V2 (HBR-313-D), and V3 (HBR-314-E) as compared to salt-treated plants. It was reported that high molecular weight protein is more accumulated in salt-tolerant varieties as compared to salt-sensitive ones in the case of barley, wheat, and rice. These salts-produced proteins are also involved in osmoregulation and production of soluble sugars [54]. Brinjal variety 4 (HBR-334-D) and V1 (ICS-BR-1351) both faced a 54% reduction in protein content as compared to control plants. V2 has a 50% reduction in protein concentration and V3 has a 36% decline in protein value at the highest salt level as compared to control plants. Similar results are where salt stress reduces proteins related to the biosynthesis of chlorophyll and downregulation of metabolic proteins in rice, sorghum, and chickpea [55]. Expression of Rubisco is reported to be inhibited under salt stress in wheat plants [56]. Total soluble sugar (TSS) of brinjal varieties is also affected in a similar way as total protein at various levels of salt stress. Plant variety 1 (ICS-BR-1351) has the highest value of TSS in the case of control plants followed by V4 (HBR-334-D), V-2 (HBR-313-D) and V-3 (HBR-314-E) as compared to salt treated plants. Salt-tolerant plants contain more accumulation of sugars and starch as upregulation of sugar content under salt stress is to lessen toxic effects [57]. However, salt caused a significant reduction in soluble proteins. Similar findings are presented by [58] in cotton where salt stress reduced the sugar content of the plant by disrupting enzymes and chlorophyll machinery. Our study demonstrated that the total amino acid content in all brinjal varieties increased as the salt concentration exceeded. V-1 (ICS-BR-1351) has maximum value of amino acid content followed by V-4 (HBR-334-D), V-2 (HBR-313-D) and V-3 (HBR-314-E). Glycophytes including tomato and alfalfa plants accumulate more amino acids and secondary metabolites in increasing salt situations for osmotic adjustment and improvement of salinity tolerance [59]. The flavonoid content of brinjal plants showed variable values as the maximum content was shown by V-3 (HBR-314-E) followed by V-2 (HBR-313-D) and V-4 (HBR-334-D). V-1 (ICS-BR-1351) showed the lowest value for flavonoid content in comparison to other varieties. However, all varieties showed a significant increase in flavonoids as salt levels increased. Following our findings stated that flavonoid is produced in salt stress as a response to stimulate biosynthesis of other metabolites to cope with the negative effect of stress. Salt-sensitive varieties produce more flavonoids as they need to adjust ROS and osmosis in salt stress. Increased accumulation of crucial phenolics i.e. flavonoids helps reduce oxidative damage in salt stress [60–62].

### Anthocyanin, lycopene, MDA, and H_2_O_2_ content

Brinjal varieties showed an increase in anthocyanin and lycopene concentration with increasing levels of salt. V-3 (HBR-314-E) had a maximum value of anthocyanin followed by V-2 (HBR-313-D) and V-4 (HBR-334-D) at 300mM salt level. More production of anthocyanin and lycopene in salt-sensitive varieties of brinjal helps plants protect against oxidative damage and nutrient imbalance [63, 64]. Application of lycopene in common bean helped plants mitigate salt stress by promoting seed germination and seedling growth [65]. In our study malondialdehyde and Hydrogen peroxide showed similar values in all brinjal varieties. All varieties showed an increase in MDA and H_2_O_2_ with increasing levels of salinity. H_2_O_2_ produced in plants under saline stress minimizes the effect of salt toxicity in eggplants as a signaling molecule that triggers protection in stressed cells [64]. also reported overproduction of MDA of almost 80% more in the brinjal plant treated with 300mM salt stress. Similar results are reported by [66] in salt-treated brinjal where an increased rate of MDA was observed to deal with salinity helping plants in salt tolerance. Our findings showed that all salt-treated plant varieties had greater value for antioxidants (peroxidase, catalase, and superoxide dismutase) as compared to control plants. Though salt-sensitive V-3 contains the maximum content of antioxidants as compared to other varieties. Previous studies on brinjal also state that antioxidants are helping agents for survival in stressed plants [67, 68, 69].

## Conclusion

In conclusion, this study highlights the significant impact of salinity on four brinjal (*Solanum melongena*) varieties. Salt stress adversely affected all varieties, with varying degrees of sensitivity observed. Morphological data consistently showed reduced shoot and root length, leaf number, root number and fresh and dry weights as salt concentration increased. V-1 (ICS-BR-1351) exhibited better growth performance, indicating some salt tolerance, while V-3 (HBR-314-E) was the most salt-sensitive. Salt stress affected photosynthetic pigments, with a decrease in chlorophyll and an increase in carotenoid levels. This suggests an adaptive response to oxidative stress caused by high salinity. Biochemical analyses revealed changes in various parameters, such as total protein, soluble sugar, flavonoids, amino acids, anthocyanin, lycopene, malondialdehyde (MDA), and hydrogen peroxide (H_2_O_2_). The alterations indicate resource allocation shifts and defense mechanisms against salt-induced oxidative damage. Antioxidant enzymes, including peroxidase, catalase, and superoxide dismutase, exhibited increased activity under salt stress, helping to counteract oxidative damage. Cluster analysis revealed complex interplays between various morphological and biochemical attributes. In summary, different brinjal varieties responded diversely to salt stress, with V-1 showing higher salt tolerance and V-3 displaying greater sensitivity. Understanding these responses is crucial for future crop management and breeding programs to enhance salt tolerance and ensure food security in saline-prone regions.

## Data Availability

The author confirms that all data generated or analyzed during this study are included in this published article.
